# Identification and validation of ubiquitination-related genes for predicting cervical cancer outcome

**DOI:** 10.3389/fgene.2025.1578075

**Published:** 2025-07-30

**Authors:** Ge Jin, Xiaomei Fan, Xiaoliang Liang, Honghong Dai, Jun Wang

**Affiliations:** ^1^ Department of Gynecology, the Fourth Hospital of Hebei Medical University, Shijiazhuang, China; ^2^ Department of Radiation Oncology, the Fourth Hospital of Hebei Medical University, Hebei Clinical Research Center for Radiation Oncology, Shijiazhuang, China

**Keywords:** ubiquitination, cervical cancer, prognosis, biomarker, bioinformatics analysis

## Abstract

**Introduction:**

Abnormalities in ubiquitination-related pathways or systems are closely associated with various cancers, including cervical cancer (CC). However, the biological function and clinical value of ubiquitination-related genes (UbLGs) in CC remain unclear. This study aimed to explore key UbLGs associated with CC, construct a prognostic model, and investigate their potential clinical and immunological significance.

**Methods:**

Differentially expressed genes (DEGs) between CC (tumor) and standard samples in self-sequencing and TCGA-GTEx-CESC datasets were identified using differential analysis. We identified overlaps between DEGs in both datasets and UbLGs, revealing key crossover genes. Subsequently, biological markers were identified via univariate Cox regression analysis and least absolute shrinkage and selection operator algorithms. After conducting independent prognostic analysis, immune infiltration analysis was performed to investigate the immune cells that differed between the two risk subgroups. Differences in immune checkpoint expression between the subgroups were analyzed. Real-Time Quantitative Polymerase Chain Reaction (RT-qPCR) was performed to confirm the expression trends of the biomarkers.

**Results:**

Differentially expressed genes related to ubiquitination were screened from the Self-seq and TCGAGTEx-CESC datasets, and five key biomarkers (MMP1, RNF2, TFRC, SPP1, and CXCL8) were identified. The risk score model constructed based on these biomarkers could effectively predict the survival rate of cervical cancer patients (AUC >0.6 for 1/3/5 years). Immune microenvironment analysis showed that 12 types of immune cells, including memory B cells and M0 macrophages, as well as four immune checkpoints, exhibited significant differences between the high-risk and low-risk groups. RT-qPCR confirmed that MMP1, TFRC, and CXCL8 were upregulated in tumor tissues.

**Discussion:**

Our study identified five ubiquitination-related biomarkers, namely, MMP1, RNF2, TFRC, SPP1, and CXCL8, which were significantly associated with CC. The validated risk model demonstrates strong predictive value for patient survival. These findings provide crucial insights into the role of ubiquitination in CC pathogenesis and offer valuable targets for advancing future research and therapeutic strategies.

## 1 Introduction

Cervical cancer (CC) is the most common cancer of the female reproductive system. The Global Cancer Statistics 2018 report indicated that approximately 577,000 newly diagnosed CC cases and 311,000 fatalities were attributed to the disease in 2018, accounting for 7.5% of all cancer-related deaths among women (Bray et al., 2018). Surgery is the primary treatment for early-stage CC, whereas concurrent chemoradiotherapy is recommended for advanced-stage CC. According to the 2018 CONCORD-3 Project report, the 5-year survival rate for CC in China (67.6%) surpassed that in the United States (62.6%) (Allemani et al., 2018). Nonetheless, approximately 30% of patients experience recurrence or metastasis following standard treatment, lacking definitive treatment, with a median survival time of 8 13 months (van Meir et al., 2014; Barter et al., 1990). Currently, the management of CC patients is transitioning toward personalized therapy, making accurate prognosis prediction crucial for individualized clinical treatment decisions.

Ubiquitin and ubiquitin-like (UB/UBL) conjugations are post-translational modifications that are crucial for nearly all biological processes, such as DNA damage repair, cell-cycle regulation, signal transduction, and protein degradation (Deshaies and Pierce, 2020). Ubiquitin is a highly conserved, heat-stable protein comprising 76 amino acids (Scott and Kleiger, 2020). Ubiquitin conjugation occurs through a three-step cascade catalyzed by three enzymes: ubiquitin-activating enzymes (E1s), ubiquitin-conjugating enzymes (E2s), and ubiquitin protein ligases (E3s) (Liu and Tan, 2020). Notably, the ubiquitin–proteasome system (UPS) degrades 80% of the intracellular proteins, thereby maintaining genomic stability and modulating signaling pathways to regulate cell proliferation and apoptosis. Abnormal expression or mutations in E3 ligases have been identified in CC, underscoring their critical role in disease onset and progression (Liu et al., 2022; Lin et al., 2019). Moreover, previous studies have explored the role of ubiquitination-related genes in other cancers. For instance, a signature comprising six ubiquitin-related genes–ARIH2, FBXO6, GNB4, HECW2, LZTR1, and RNF185–was developed to predict the biochemical recurrence of prostate cancer (Song et al., 2021). However,in the context of cervical cancer, the collective impact of multiple ubiquitination-related genes remains underexplored. This study aims to fill this gap by identifying and validating key ubiquitination-related genes that are significantly associated with cervical cancer outcomes.

The aim of the present study was to identify ubiquitination-related biomarkers in CC using self-collected transcriptomic data and public databases through differential analysis (Love et al., 2014), univariate Cox analysis, least absolute shrinkage and selection operator (LASSO) analysis, and other bioinformatics methods. Additionally, we conducted enrichment and immune-infiltration analyses within high- and low-risk groups to shed light on CC pathogenesis, diagnosis, and treatment (Heagerty et al., 2000; Yu et al., 2012; Wu et al., 2021; Ito and Murphy, 2013).

## 2 Manuscript formatting

### 2.1 Data sources

In the present study, we performed RNA sequencing (Seq) of eight human cervical cancer (CC) tissue samples and their adjacent non-cancerous tissue samples to form a self-seq dataset. The Ethics Committee of the Fourth Hospital of Hebei Medical University, China, approved this retrospective research (approval number: 2023KS014). Written informed consent was obtained from the participants.

To enhance the accuracy of our research, we obtained additional CC expression profile data from the UCSC Xena database, which integrates data from The Cancer Genome Atlas-Cervical Squamous Cell Carcinoma (TCGA-CESC) project, including 304 CC samples and three normal samples, as well as 10 normal samples from the GTEx project. These data constituted the TCGA-GTEx-CESC dataset, which comprised 304 tumor samples and 13 normal samples. To validate our research findings, we used the GSE52903 dataset from the Gene Expression Omnibus (GEO) database, which included 55 tumor samples and 17 normal samples. Based on a search of the GeneCards database using the keyword “Ubiquitin-like modifiers”, genes with a score ≥3 were filtered, ultimately identifying 465 ubiquitination-related genes (UbLGs) (Supplementary Table S1), laying the foundation for an in-depth study of the molecular mechanisms of CC and exploration of potential therapeutic targets.

### 2.2 RNA extraction, library construction, and data processing

Total RNA was extracted and purified from the samples using TRIzol reagent, in accordance with the manufacturer’s instructions. A NanoDrop ND-1000 spectrophotometer (NanoDrop, Wilmington, DE, USA) was used to evaluate the quantity and purity of RNA. Agarose gel electrophoresis was used to confirm RNA integrity. The RNA was then fragmented and reverse-transcribed into cDNA. The double-stranded synthesis process was carried out utilizing RNase H (NEB, cat.m0297, USA) and *E. coli* DNA polymerase I (NEB, cat.m0209, USA). The final cDNA library contained inserts with an average size of 300 ± 50 bp. Following standard protocols, an Illumina NovaSeq 6000 (LC-Biotechnology CO., Ltd., Hangzhou, China) was used for sequencing. Sequencing data were aligned to the human reference genome (GRCh38.105) to ensure expression quantification and quality control, resulting in a gene count expression matrix for each transcriptome group.

### 2.3 Acquisition and enrichment analysis of crossover genes

The DESeq2 (v 1.36.0) package was used to identify differentially expressed genes (DEGs) between standard samples and tumor samples in the Self-seq and TCGA-GTEx-CESC datasets (p-value <0.05 & |log2Fold Change| > 0.5). The pheatmap (v 1.0.12) and ggplot2 (v 3.4.1) packages were used to create heat maps and volcano plots of these DEGs, respectively. Upregulated DEGs in both datasets and UbLGs were overlaid to identify the upregulated crossover genes. Similarly, the downregulated DEGs and UbLGs were analyzed to identify the downregulated crossover genes. These crossover genes were used for the subsequent analyses. The clusterProfiler (v 4.6.2) package (p.adjust <0.05 & count >2) was used to conduct Gene Ontology (GO) and Kyoto Encyclopedia of Genes and Genomes (KEGG) enrichment analyses to further probe the biological functions and signaling pathways linked to the crossover genes.

### 2.4 Development and validation of the prognostic model

First, feature genes were identified using univariate Cox analysis of the crossover genes (p < 0.05). Thereafter, LASSO and Cox regression models based on these feature genes (family = Cox) were used to identify the biomarkers. Thirteen tumor samples with incomplete information from the TCGA-CESC dataset were excluded, leaving 291 samples. A 7:3 ratio was used to divide the samples into testing and training sets (203:88). A risk model was generated based on biomarker expression. The training, testing, and GSE52903 validation sets were categorized into high- and low-risk groups as per the optimal threshold value of the risk score (risk score = ∑_1^n coef (genei)*expression (genei)). Kaplan-Meier (K-M) survival curves and receiver operating characteristic (ROC) curves (1-, 3-, and 5-year) were generated. Subsequently, the concordance function in the “survival” package (v 3.5-3) (Lei et al., 2023) was utilized to calculate the C-index, which was used to measure the consistency of the model in ranking the survival of samples. Further risk model validation was conducted on the testing set and GSE52903 validation set to assess its suitability.

### 2.5 Independent prognostic analysis

The risk score and clinicopathological factors (stage, race, grade, age, and pathology (N, T, M)) of the 203 tumor samples in the training set were subjected to univariate Cox analysis. This was followed by proportional hazard assumption and multivariate Cox analysis of the identified clinical features to determine independent prognostic indicators. These parameters were employed to generate a nomogram that predicted CC patient survival rates over 1,3, and 5 years, and the accuracy of the nomogram was validated using calibration curves. Meanwhile, the ROC curve was plotted using the “survminer” package (version 0.4.9) (Li and Lyu, 2025) to evaluate the diagnostic value of the nomogram.

### 2.6 The enrichment analyses and immune microenvironment analysis

Differential analysis was conducted between the high- and low-risk subgroups, sorting expression profile genes by fold change. The GO and KEGG background gene sets were utilized to execute gene set enrichment analysis (GSEA) (p.val <0.05, |NES| > 1, q.val <0.2). Using the psych (v 2.1.6) package (Robles-Jimenez et al., 2021), Spearman’s correlation coefficients between key genes and other genes in the training set samples were calculated, and genes were ranked in descending order based on these coefficients. GSEA was performed using the clusterProfiler (v 4.6.2) package (Suo et al., 2022) with the same threshold, and the top 10 significant pathways with p.values less than 0.05 were identified. The infiltration levels of immune cells between the two risk groups were verified using the CIBERSORT method, and the Wilcoxon test was used to compare differential immune cells. Spearman’s method was used to compare the associations between biomarkers and risk scores for differentially expressed immune cells.

### 2.7 Immune checkpoints and immune-response analyses

The Wilcoxon test was used to compare differences in routine immune checkpoints (galectin 9 (GAL9), LGALS9, CTLA-4 (CTLA4), and TIGHT), TIM-3(HAVCR2), PD-L1(CD274), LAG-3 (LAG3), PD-L2 (PDCD1LG2), and PD-1 (PDCD1) between the two subgroups. The Spearman’s method was used to determine the association between immune checkpoints and risk scores. The cycle STEP 1-7 immune activity scores were obtained using Tracking Tumor Immunophenotype (TIP) online tools to compare the anti-cancer immune response scores between the two risk groups.

### 2.8 Drug sensitivity analysis

To further investigate the differences in chemotherapy responses between different risk groups, the pRRophetic package (v0.5) (Lu et al., 2019) was used to calculate the half-maximal inhibitory concentration (IC50) values of 138 commonly used chemotherapeutic and targeted drugs in patient samples from both groups. The Wilcoxon rank-sum test was employed to compare the IC50 values of the screened drugs between the two groups, and the top 20 differential compounds were visualized using box plots. The chemical structures of all compounds were retrieved from the PubChem database (https://pubchem.ncbi.nlm.nih.gov/).

### 2.9 RT-qPCR validation

To verify the expression of the biomarkers, the Wilcoxon test was used to validate the expression differences of prognostic genes in the TCGA-GTEx-CESC dataset, and the external validation set GSE52903. After approval from the Ethics Committee of the Fourth Hospital of Hebei Medical University, we obtained patient consent and collected frozen tissue samples, including five tumor and five standard samples. Total RNA was isolated using TRIzol (Ambion), and reverse transcription was performed with the SureScript-First-strand-cDNA-synthesis-kit (Servicebio). The cDNA was diluted 5–20 times with ddH2O (RNase/DNase-free). PCR amplification was conducted on the CFX96 RT-qPCR instrument under the following conditions: 95°C for 1 min (pre-denaturation), followed by 40 cycles of 95°C for 20 s (denaturation), 55°C for 20 s (annealing), and 72°C for 30 s (elongation). The expression levels of the biomarkers were normalized to the housekeeping gene glyceraldehyde-3-phosphate dehydrogenase (GAPDH). The Wilcoxon signed-rank test was used to compare the expression levels of the biomarkers between the tumor and normal groups. The primer sequences used in the present study are listed in Table 1.

**TABLE 1:** | Correlated primer sequence.

Primer	Sequence
MMP1 F	AGA​AAG​AAG​ACA​AAG​GCA​AGT​TGA
MMP1 R	AAA​CTG​AGC​CAC​ATC​AGG​CA
RNF2 F	GCA​GCT​GAT​ACC​AGA​GTC​TTG​C
RNF2 R	GCC​TCC​TGA​GGT​GTT​CGT​TG
TFRC F	GGC​TAC​TTG​GGC​TAT​TGT​AAA​GG
TFRC R	CAG​TTT​CTC​CGA​CAA​CTT​TCT​CT
SPP1 F	CGG​GGG​TTC​CGT​TAT​CAT​GT
SPP1 R	TTT​CTC​ATC​CTC​CCT​CCG​GT
CXCL8 F	ACC​CCA​AGG​AAA​ACT​GGG​TG
CXCL8 R	GGT​CAT​GAG​TAC​AAC​AAA​CTC​ACT
internal reference-GAPDH F	CGA​AGG​TGG​AGT​CAA​CGG​ATT​T
internal reference-GAPDH R	ATG​GGT​GGA​ATC​ATA​TTG​GAA​C

## 3 Results

### 3.1 Sequencing outcomes

The base quality Q30 of each sample exceeded 90%, indicating favorable sequencing results (Table 2). Additionally, the comparison rate for each sample was above 90%, demonstrating satisfactory sequencing quality, suitable for further analysis (Table 3).

**TABLE 2:** | Quality analysis of sequencing data.

Sample	Raw Reads	Clean Reads	Raw Base(G)	Clean Base(G)	Effective Rate(%)	Q20	Q30	GC Content(%)
SJZ8C1	42293682	40891570	6.34	6.07	96.6848	5.97 (98.40%)	5.74 (94.58%)	51.38
SJZ8C2	46897424	44355056	7.03	6.57	94.5789	6.44 (98.02%)	6.14 (93.57%)	50.07
SJZ8C3	39590408	38101116	5.94	5.66	96.2383	5.58 (98.59%)	5.38 (95.08%)	50.41
SJZ8C4	43195342	41893498	6.48	6.21	96.9861	6.11 (98.31%)	5.86 (94.29%)	50.17
SJZ8C5	53821444	51971168	8.07	7.7	96.5622	7.57 (98.28%)	7.25 (94.19%)	48.49
SJZ8C6	43878988	42098858	6.58	6.23	95.9431	6.12 (98.22%)	5.86 (94.11%)	50.6
SJZ8C7	49271854	47856430	7.39	7.11	97.1273	7.00 (98.51%)	6.74 (94.89%)	51.93
SJZ8C8	45959454	44394656	6.89	6.59	96.5953	6.50 (98.60%)	6.27 (95.13%)	51.16
SJZ8N1	39302096	36848794	5.9	5.44	93.7578	5.32 (97.85%)	5.06 (93.10%)	49.96
SJZ8N2	36962998	34697374	5.54	5.11	93.8706	4.99 (97.66%)	4.73 (92.63%)	49.11
SJZ8N3	39889356	37657738	5.98	5.58	94.4055	5.47 (98.11%)	5.23 (93.81%)	50.36
SJZ8N4	38900418	36614314	5.84	5.41	94.1232	5.31 (98.10%)	5.08 (93.85%)	54.18
SJZ8N5	55256312	52506492	8.29	7.77	95.0235	7.61 (97.85%)	7.24 (93.14%)	51.08
SJZ8N6	39385488	37983072	5.91	5.61	96.4393	5.48 (97.70%)	5.21 (92.78%)	50.34
SJZ8N7	51499052	49305362	7.72	7.32	95.7403	7.22 (98.58%)	6.96 (95.07%)	51.47
SJZ8N8	44799836	43234238	6.72	6.41	96.5053	6.31 (98.37%)	6.06 (94.50%)	51.25

**TABLE 3:** | Reference sequence alignment analysis.

Sample	Input reads	Mapped reads	Mapping ratio
SJZ8C1	43056530	42003995	97.56%
SJZ8C2	46877727	45572206	97.22%
SJZ8C3	40397445	39308050	97.30%
SJZ8C4	44373936	43077516	97.08%
SJZ8C5	55042903	53777564	97.70%
SJZ8C6	45672499	44136941	96.64%
SJZ8C7	51281178	49937064	97.38%
SJZ8C8	46714568	45653136	97.73%
SJZ8N1	39388091	37895543	96.21%
SJZ8N2	36670056	35593222	97.06%
SJZ8N3	39788616	38941292	97.87%
SJZ8N4	41732983	39909617	95.63%
SJZ8N5	56693500	55319863	97.58%
SJZ8N6	40382882	39172352	97.00%
SJZ8N7	52875606	51247061	96.92%
SJZ8N8	46786270	44748307	95.64%

### 3.2 Identification of cervical cancer-related DEGs

**FIGURE 1:**
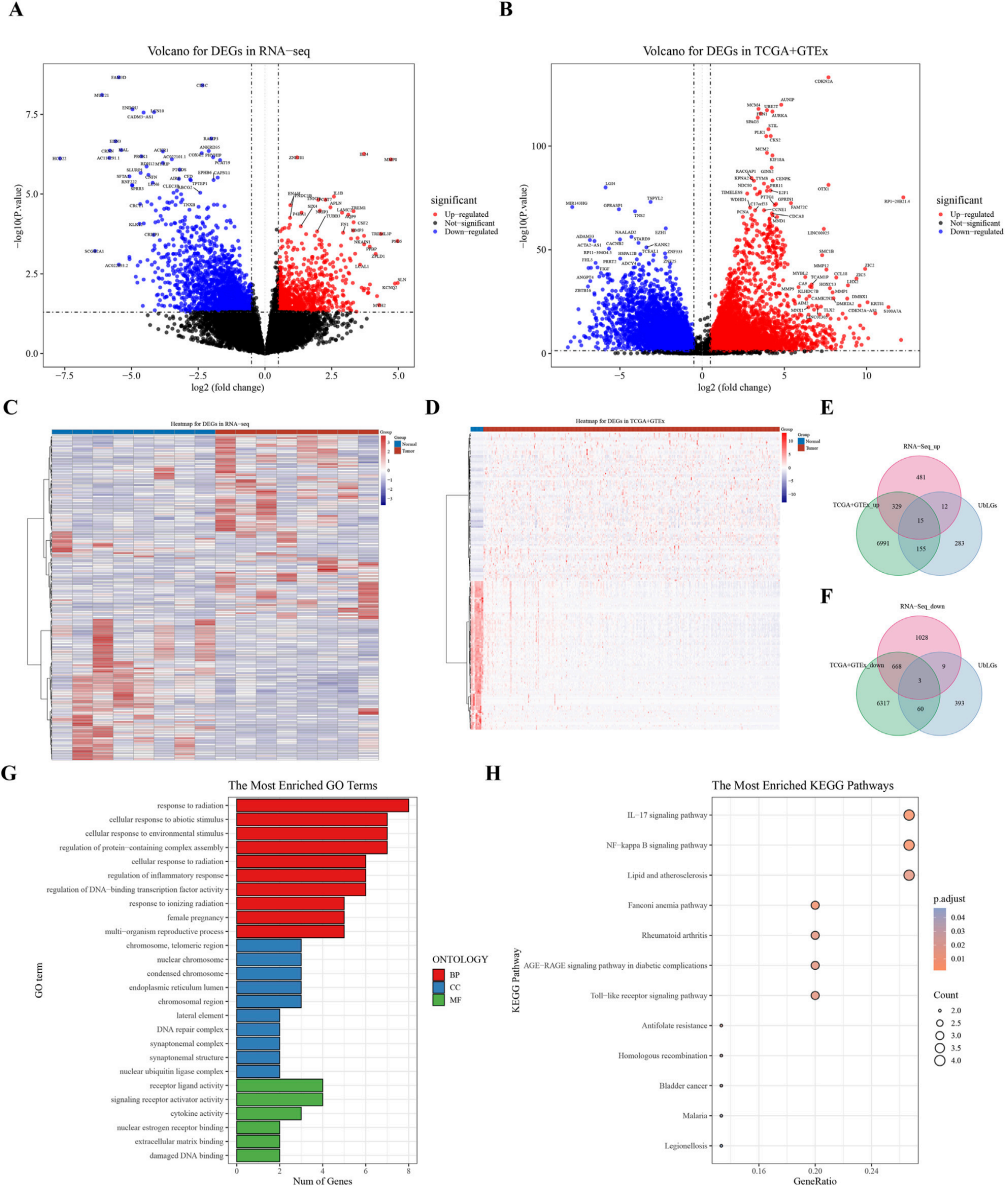
Analysis of Differentially Expressed Genes (DEGs) in Cervical Cancer. **(A)** Volcano plot of DEGs derived from RNA sequencing data. **(B)** Volcano plot of DEGs from the combined TCGA and GTEx datasets. **(C, D)** Heat maps showing the expression profiles of identified DEGs. **(E, F)** Venn diagram representing the overlap of DEGs, highlighting 18 key crossover genes. **(G, H)** GO and KEGG pathway analysis of the crossover genes, providing insights into their biological functions and associated pathways.

A total of 465 ubiquitination-related genes (UbLGs) were identified from the GEO dataset and used as a reference to explore their potential involvement in cervical cancer. Our study identified 2545 and 14,538 DEGs between tumor samples and standard samples in the Self-seq and TCGA-GTEx-CESC datasets, respectively (Figures 1A,B; Supplementary Tables S2, 3). The expression levels of these DEGs are depicted in heat maps (Figures 1C,D). Intersection analysis revealed 18 genes that were significantly differentially expressed, including the five selected biomarkers (MMP1, RNF2, TFRC, SPP1, and CXCL8), which are significantly associated with cervical cancer progression (Figure 1E,F). Among these, MMP1 and RNF2 are particularly noteworthy because of their close association with cervical cancer tumorigenesis, particularly in terms of invasiveness and metastasis. The role of MMP1 in modulating the tumor microenvironment (TME) has been well documented, and RNF2, which functions as an E3 ubiquitin ligase, plays a crucial role in regulating cell-cycle progression and the DNA damage response. The discovery of these genes offers crucial insights into the molecular underpinnings of cervical cancer and potential identification of novel therapeutic targets. Enrichment analysis linked these genes to GO items such as ‘response to radiation’ and KEGG pathways, including the ‘IL-17 signaling pathway’ (Figures 1G,H; Supplementary Tables S4, S5).

### 3.3 Identification of five biomarkers

**FIGURE 2:**
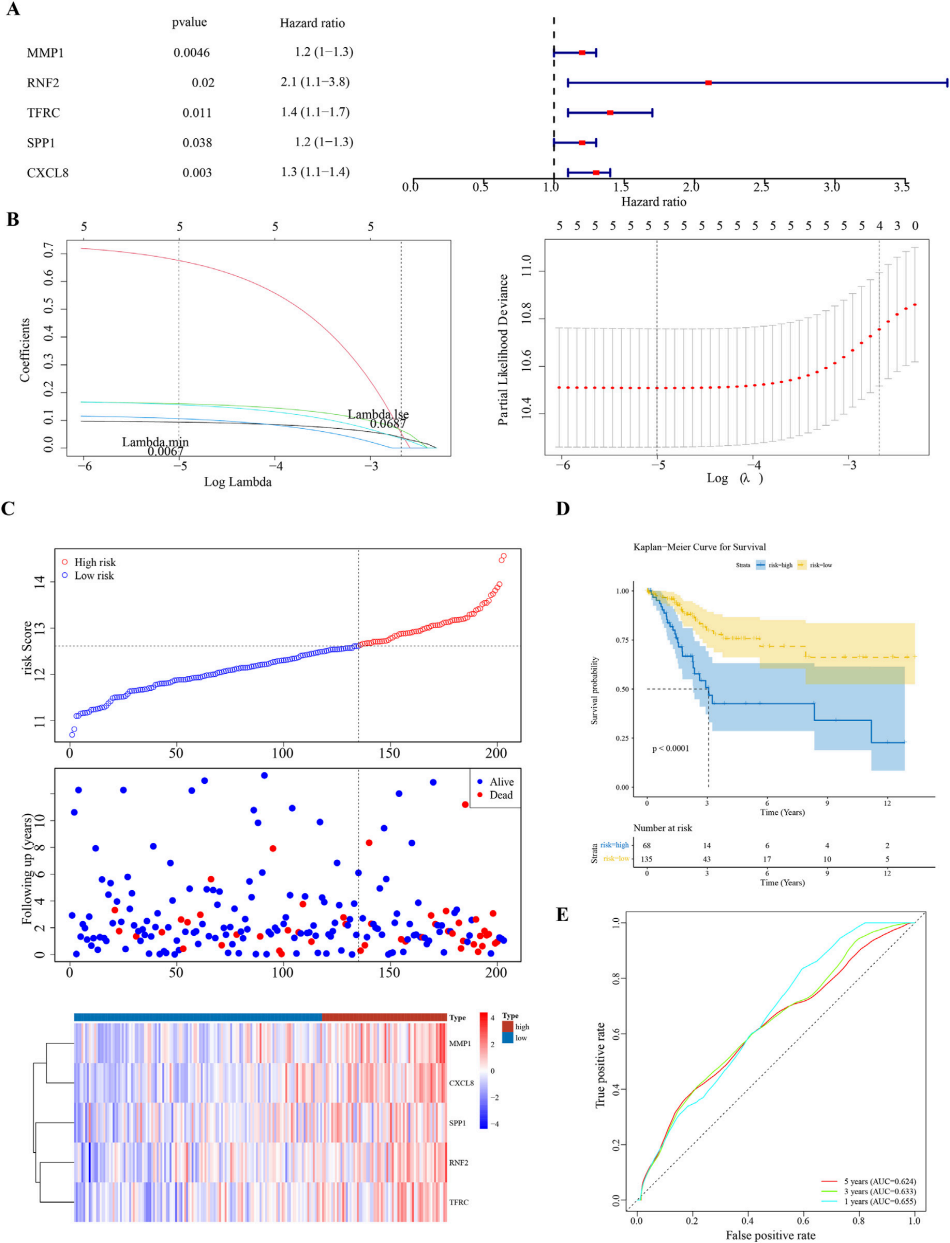
Prognostic Biomarker Identification and Survival Analysis **(A)** Univariate Cox regression analysis identifying five significant biomarkers (MMP1, RNF2, TFRC, SPP1, CXCL8). **(B)** LASSO and Cox regression model for biomarker selection. **(C)** Distribution of cervical cancer samples into high-risk and low-risk groups based on biomarker expression levels. **(D)** Kaplan-Meier survival curves demonstrating survival differences between high-risk and low-risk groups. **(E)** Receiver Operating Characteristic (ROC) curves for 1-, 3-, and 5-year survival predictions, assessing the model’s predictive accuracy.

**FIGURE 3:**
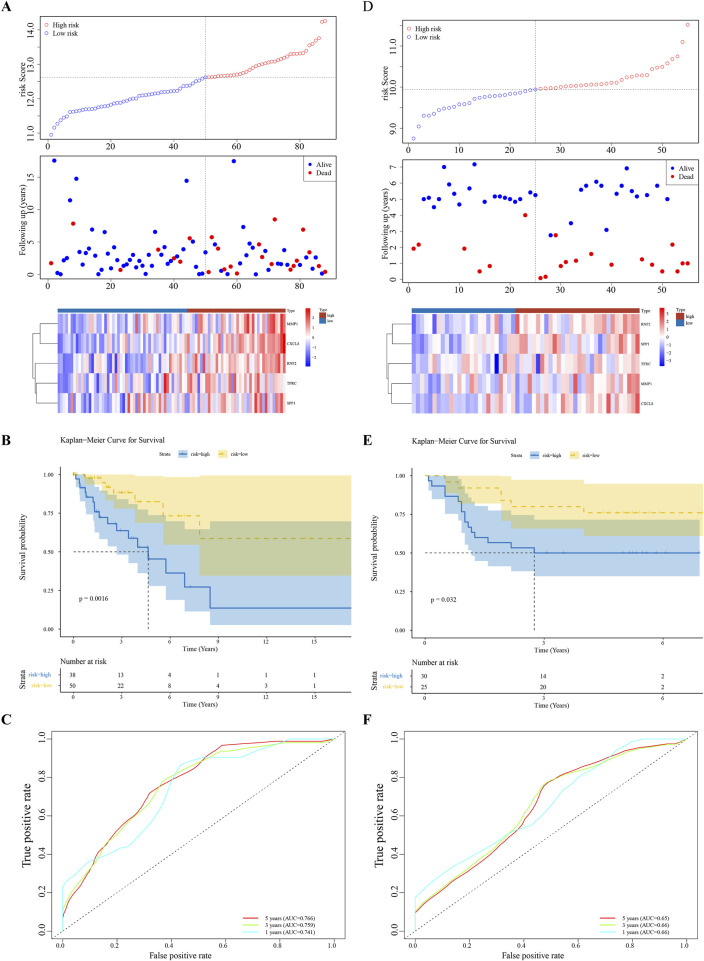
Model Validation Across Datasets **(A–F)** Consistency of the prognostic model’s performance in the testing set and the GSE52903 validation set, confirming the model’s reliability and generalizability.

Univariate Cox regression analysis identified five feature genes (MMP1, RNF2, TFRC, SPP1, and CXCL8) as biomarkers (Figure 2A). These were further validated using the LASSO and Cox regression models (lambda. min, 0.0067) (Figure 2B). The CC samples in the training set were categorized into high-(68 samples) and low-risk (135 samples) groups using a threshold value of 12.61. The high-risk group exhibited higher expression levels of the biomarkers (Figure 2C). Notably, significant differences in survival were noted between these groups (p < 0.05) (Figure 2D). The C-index of the risk model was 0.691, and the area under the ROC curve (AUC) for the 1-, 3-, and 5-year predictions exceeded 0.6, confirming the prognostic value of the model (Figure 2E). The efficacy of the model was consistent across both the testing and GSE52903 validation sets. We selected genes with a p-value less than 0.05 as candidate genes significantly associated with survival outcomes. This threshold is a standard choice in the field and aids in controlling the false-positive rate. We employed cross validation to determine the optimal lambda value, with a final lambda. min of 0.0067, to balance the bias and variance of the model. To construct the prognostic model, we generated ROC curves for 1-year, 3-year, and 5-year survival predictions, which included the corresponding confidence intervals, to assess the predictive performance of the model (Figures 3A–F).

### 3.4 Creation of nomogram

**FIGURE 4:**
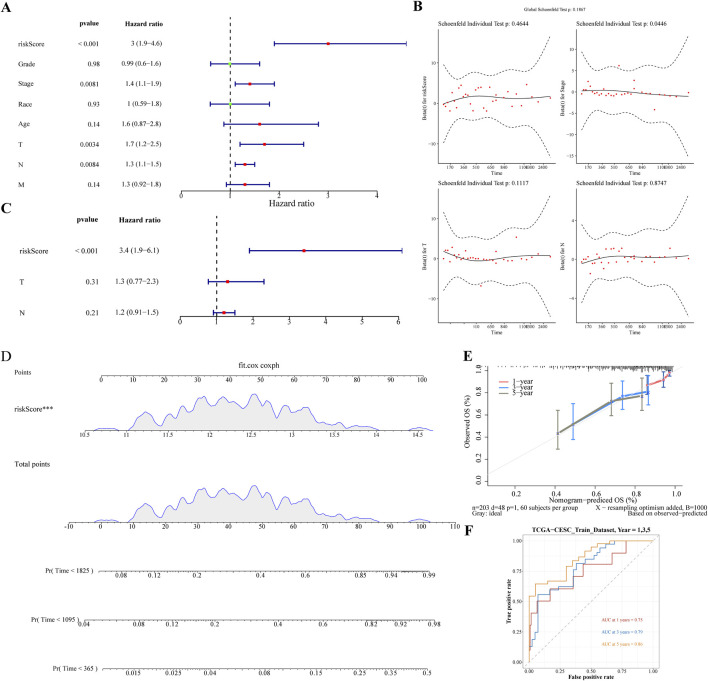
Nomogram Development for Prognostic Prediction **(A)** Univariate Cox analysis identifying significant clinical factors including Risk Score, Stage, and Pathological T and N stages. **(B)** Figure of schoenfeld residual test **(C)** Risk Score confirmed as an independent prognostic factor. **(D)** Nomogram constructed based on the risk score to predict 1-, 3-, and 5-year survival rates for cervical cancer patients. **(E)** Calibration curves of nomogram. **(F)** The ROC curve of the nomogram.

The Risk score, Stage, and Pathological T and N stages were identified as significant using univariate Cox analysis (Figure 4A). These clinical factors conformed to the proportional risk hypothesis (Figure 4B), with risk scores emerging as independent prognostic factors (Figure 4C). A nomogram based on the risk score was developed to forecast the survival rates of CC patients (1-, 3-, and 5-year), with a C-index of 0.692, and the calibration curve demonstrating the predictive accuracy of the model (Figure 4D,E). Additionally, the ROC curve showed AUC values of 0.77, 0.78, and 0.75 for 1/3/5 years, further demonstrating that the nomogram model had high discriminative ability and good predictive performance (Figure 4F).

### 3.5 The GSEA in two risk subgroups

To gain deeper insights into the roles of the five selected biomarkers in cervical cancer, we conducted GSEA. Our analysis unveiled that these biomarkers are significantly enriched in pathways tied to protein ubiquitination and degradation, underscoring their strong association with ubiquitination processes (Supplementary Figure S1). In the context of our study, GSEA was employed to investigate the functional enrichment of differentially expressed genes (DEGs) associated with high- and low-risk subgroups of CC patients, as identified using our prognostic model. Our analysis revealed significant enrichment of specific GO terms and KEGG pathways that were differentially represented between the two risk groups. Notably, the ‘Cornified Envelope’ GO term was significantly enriched in the high-risk group, suggesting an overrepresentation of genes related to the formation of a protective layer in epithelial tissues, which is often associated with keratinization. This finding implied a role for these genes in the development and progression of cervical cancer, potentially influencing the interaction of the tumor with the host immune system and its response to therapeutic interventions.

**FIGURE 5:**
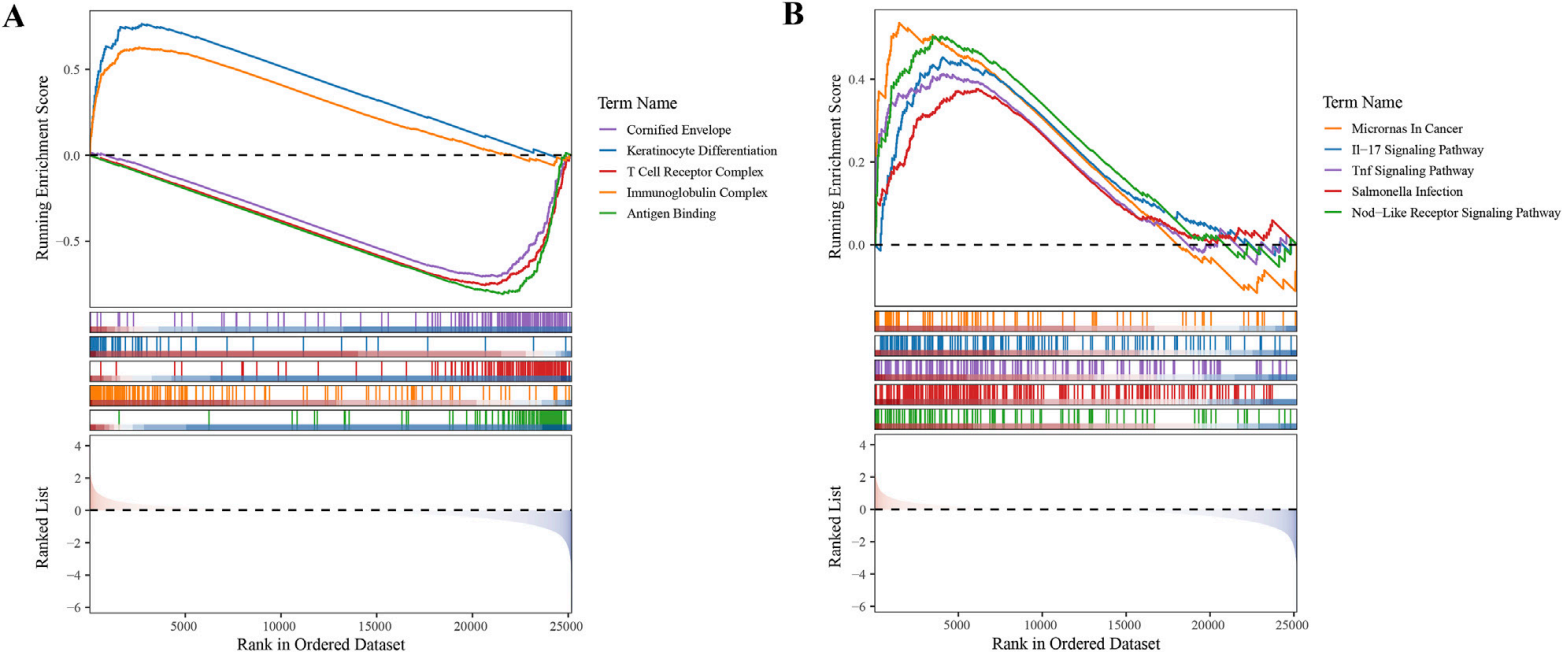
Gene Set Enrichment Analysis (GSEA) in High- and Low-Risk Subgroups **(A)** GO term enrichment analysis revealing biological processes overrepresented in high- and low-risk subgroups. **(B)** KEGG pathway enrichment analysis highlighting signaling pathways differentially active between the two risk groups.

Additionally, the IL-17 signaling pathway emerged as a significantly enriched KEGG pathway. The IL-17 pathway plays a crucial role in the inflammatory response and has been implicated in the pathogenesis of various cancers, including cervical cancer. The IL-17 family of cytokines promotes tumor growth, survival, angiogenesis, and immune evasion, thereby contributing to a more aggressive tumor phenotype. The enrichment of this pathway in our high-risk group suggests that IL-17-mediated signaling may be associated with poor prognosis in patients with cervical cancer, possibly through the recruitment of immunosuppressive cells and the promotion of an immunosuppressive TME (Figures 5A,B).

In conclusion, our GSEA findings provided valuable insights into the molecular mechanisms underlying the differential prognosis of CC patients. The identification of specific biological processes and pathways enriched in high- and low-risk patient groups offers a foundation for future research aimed at developing targeted therapies and improving patient outcomes (Supplementary Tables S6,7).

### 3.6 Immune-infiltration analysis between two risk subgroups

**FIGURE 6:**
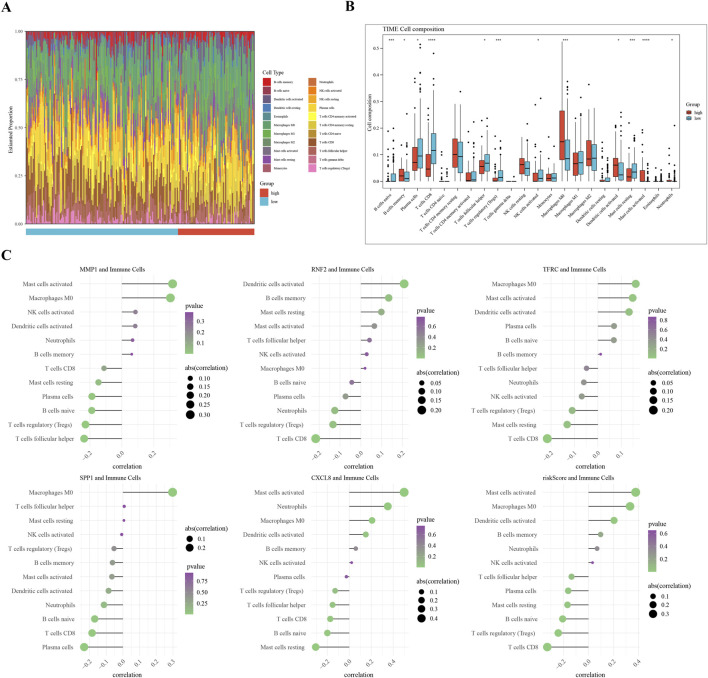
Immune Infiltration Analysis in Cervical Cancer Subgroups **(A)** Overview of differential immune cell populations between high-risk and low-risk subgroups. **(B)** Bar plot of twelve differentially abundant immune cells, showing fold change in abundance. **(C)** Visualization of Spearman’s correlation coefficients between risk scores and expression levels of differential immune cells, indicating the strength and significance of correlations.

A comparative analysis of the two groups in terms of immune cell abundance was performed (Figure 6A). Twelve differentially expressed immune cell types were identified, including memory B cells and Macrophages M0 (Figure 6B). RNF2 is negatively associated with CD8 + T cells, whereas MMP1 is positively associated with activated mast cells. The highest correlation with activated mast cells was observed for the risk score (Figure 6C). We observed significant differences in immune cell subsets that were closely associated with prognosis. Notably, higher levels of neutrophils, macrophages, CD4 + T cells, NK cells, and B cells in the low-risk group may correlate with a better prognosis. These findings are consistent with those of previous studies, indicating that the infiltration and functional status of immune cells are important predictive factors for cervical cancer prognosis.

### 3.7 Analyses of immune-checkpoint expression and anti-cancer immune response

**FIGURE 7:**
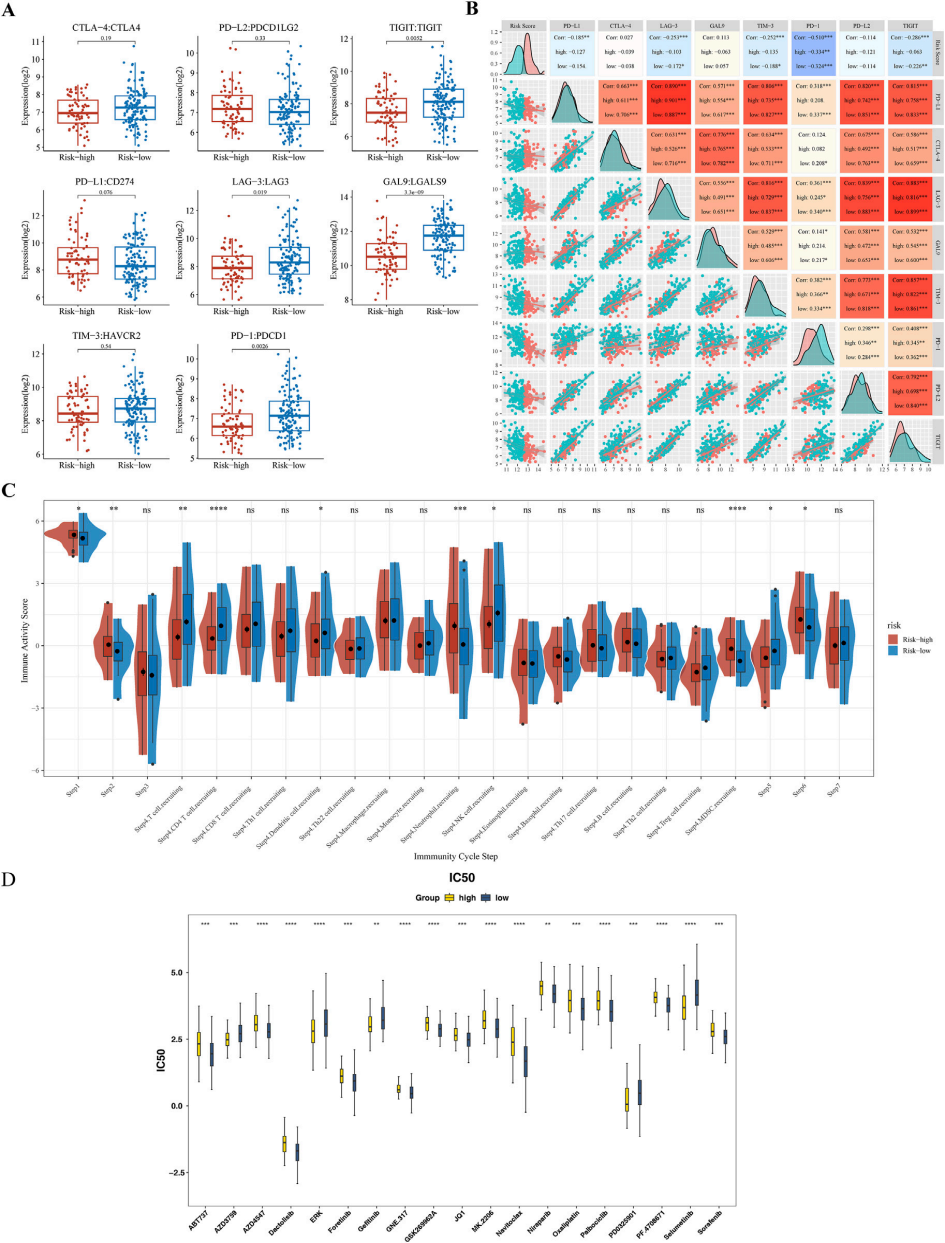
Immune Checkpoint Expression and Anti-cancer Immune Response Analysis **(A)** Comparative analysis of immune checkpoint molecule expression levels (TIGIT, LAG-3, GAL9, PD-1) between high-risk and low-risk subgroups. **(B)** Spearman’s correlation analysis between risk scores and PD-1 expression levels. **(C)** Differential immune activity scores from the TIP tool, comparing the anti-cancer immune response in high-risk versus low-risk groups. **(D)** Box plot of drug sensitivity differences between high-risk and low-risk groups. ** represented P < 0.01, *** represented P < 0.001, **** represented P < 0.0001.

Significant differences in the expression of the immune checkpoints T-Cell Immunoreceptors with Ig and ITIM domains (TIGIT), Lymphocyte Activation Gene-3 (LAG-3), Galectin-9 (GAL9), and Programmed Death-1 (PD-1) were observed between the subgroups (p < 0.05) (Figure 7A). Contrary to classical associations with T-cell exhaustion and poor prognosis, these markers were significantly elevated in the “low-risk” subgroup. PD-1 expressio correlated strongly with the risk score (|Cor| > 0.3) (Figure 7B). The TIP analysis further identified ten differential immune activity scores, including Step 4 CD4 T cells and Step 4 neutrophils (Figure 7C). This paradoxical elevation of immune checkpoints in low-risk patients coincided with higher infiltration of memory B cells and CD4^+^ T cells (Figure 6B), suggesting a context where checkpoint expression may reflect active immune engagement rather than exhaustion.

### 3.8 Drug sensitivity

Drug sensitivity testing achieved personalized cancer treatment regimens by matching pharmacological responses with tumor-specific gene profiles, thereby improving therapeutic efficacy and safety while reducing adverse reactions. A comparative analysis of the IC50 values of 138 compounds found that a total of 71 drugs showed statistically significant differences (p < 0.05) (Supplementary Table S8). Among them, in the top 20 drugs, the IC50 values of 14 compounds (such as Dactolisib, Oxaliplatin, Sorafenib, etc.) in the high-risk group increased, while the IC50 values of six compounds (such as Gefitinib, Selumetinib, etc.) decreased (Figure 7D).

### 3.9 Expression and RT-qPCR validation of biomarkers

**FIGURE 8:**
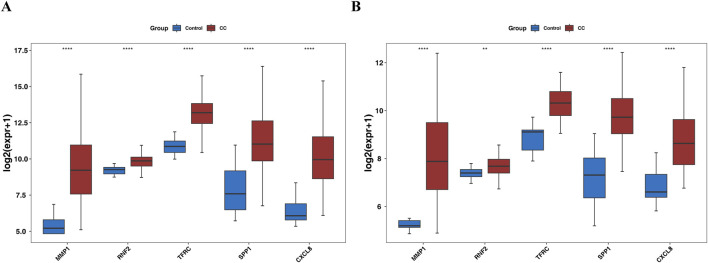
Box plot for expression validation of biomarkers **(A)** the TCGA-GTEx-CESC dataset. **(B)** GSE52903 dataset.

Gene expression analysis further showed that in the TCGA-GTEx-CESC dataset, and GSE52903, the expression levels of MMP1, RNF2, TFRC, SPP1, and CXCL8 were significantly upregulated in the CC group compared with the control group (P < 0.05) (Figures 8A,B). In our study, RT-qPCR validation was performed on five tumor samples and five normal samples. The expression levels of MMP1, TFRC, and CXCL8 were significantly upregulated in the tumor group compared to the normal group (p < 0.05). However, no significant differences were observed in the expression levels of RNF2 and SPP1 between the two groups (p > 0.05). The Wilcoxon signed-rank test was used to compare the expression levels of the biomarkers between the tumor and normal groups. The expression levels were normalized to those of the housekeeping gene GAPDH, which served as an internal control to account for variations in sample quality and the amount of total RNA added to the reactions.

**FIGURE 9:**
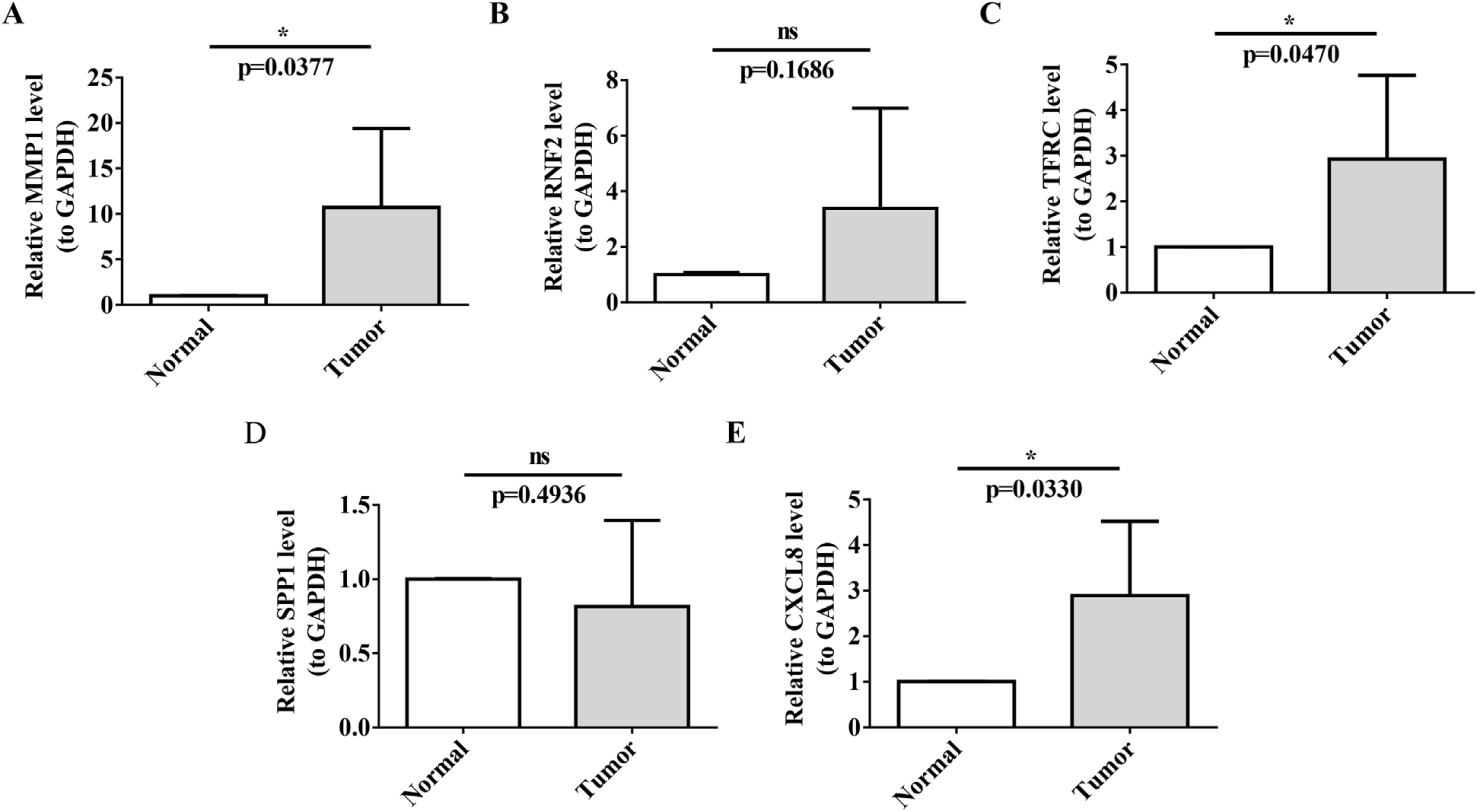
RT-qPCR Validation of Prognostic Biomarkers **(A–E)** Validation of biomarker expression (MMP1, TFRC, CXCL8) in cervical cancer tissues versus normal paracancerous tissues by RT-qPCR. Each panel displays relative expression levels, with sample types indicated on the x-axis and fold change in expression on the y-axis. Asterisks denote statistical significance levels, with ns indicating non-significance and *P < 0.05 indicating significant expression differences.

Our RT-qPCR results confirmed the upregulation of MMP1, TFRC, and CXCL8 in the tumor group, which was consistent with the findings of our previous analyses (Figures 9A,C,E).

In contrast, the expression of SPP1 did not show a significant difference between tumor tissues and normal tissues in our RT-qPCR analysis (p-value not significant, ns), which may indicate that its role in cervical cancer is less pronounced or that its expression is regulated at the post-transcriptional level.

Quantitative real-time polymerase chain reaction (RT-qPCR), a technique renowned for its sensitivity and specificity, was used to quantify and validate the expression levels of key biomarkers in cervical cancer tissues compared with those in normal paracancerous tissues. By normalizing these levels to those of the housekeeping gene GAPDH, we ensured the accuracy of our gene-expression measurements, accounting for variations in sample quality and RNA quantity.

RT-qPCR analysis confirmed the significant upregulation of MMP1, TFRC and CXCL8 in tumor tissues, consistent with our previous differential gene-expression studies. These findings underscore the potential of these biomarkers in cervical cancer, suggesting their involvement in tumor progression and their value as therapeutic targets or prognostic indicators.

Conversely, the expression of SPP1 was not significantly different between tumor tissues and normal tissues, as indicated by the non-significant p-value in the RT-qPCR analysis. This implied that the role of SPP1 in cervical cancer may be less direct or may be subject to post-transcriptional regulation, warranting further investigation into its functional significance.

Furthermore, RT-qPCR analysis did not reveal a significant upregulation of Ring Finger Protein 2 (RNF2) in the tumor group, with a p-value of 0.1686, which was above the threshold for statistical significance. This suggests that its involvement in cervical cancer might not be primarily through mRNA-level changes or that it could be regulated at the post-transcriptional or post-translational levels. The lack of significant mRNA upregulation does not preclude the potential role of RNF2 in cervical cancer; it may be involved in mechanisms such as protein stability and subcellular localization.

In conclusion, although RNF2 did not show significant mRNA upregulation in our RT-qPCR analysis, its potential as a therapeutic target or prognostic indicator for cervical cancer should not be overlooked. Further research is necessary to explore the functional significance of RNF2 in tumorigenesis, invasion, metastasis, and interactions with other molecular pathways. Investigating RNF2 protein levels and activity in cervical cancer could yield valuable insights into its broad implications in the molecular landscape of the disease (Figures 9B,D).

## 4 Discussion

CC is often linked to persistent hrHPV exposure and remains challenging despite advancements in surgery, chemoradiation, anti-angiogenic therapy, and immunotherapy (Morris, 2019; Hass et al., 2017; Nishio et al., 2021; Tewari et al., 2014). The quest for precise biomarkers is essential for early detection and monitoring of disease progression.

In this study, we identified five ubiquitination-related biomarkers (MMP1, RNF2, TFRC, SPP1, and CXCL8) that are significantly associated with cervical cancer. These biomarkers enhance our understanding of the molecular mechanisms of cervical cancer and provide potential targets for diagnosis, prognosis, and therapy. However, our study has some limitations that need to be considered when interpreting the results.

We used a local self-seq of eight pairs of cervical cancer and adjacent non-cancerous tissues to explore potential biomarkers. Although this dataset is small, it offers a pilot foundation for identifying promising leads. Importantly, our key findings from the self-seq dataset were validated against the larger TCGA-GTEx-CESC dataset (304 tumor samples and 13 normal samples) and the GSE52903 dataset (55 tumor samples and 17 normal samples). The consistency across these datasets strengthens the reliability of our results and reduces concerns about the smaller sample size of the self-seq data. Moving forward, we plan to expand our sample collection and combine our data with additional public resources to further refine and validate our findings. We believe this work provides a solid basis for future research aiming to improve the understanding and treatment of cervical cancer.

One significant limitation of our study is the absence of the concordance index (C-index) in our analysis. Due to data limitations, particularly sample size and the availability of follow-up data, we were unable to calculate and report the C-index, which is a widely recognized measure for evaluating the predictive accuracy of survival models. Future studies with larger sample sizes and more detailed follow-up information are needed to further validate our findings and to provide a more comprehensive evaluation of the model’s performance, including the calculation of the C-index.

The validation of the five gene signatures was based on a relatively small number of tissue samples (n = 5) and was conducted solely at the RNA level. Given the involvement of these genes in post-translational regulation, further validation at the protein level is necessary to comprehensively assess their expression and functional impact in cervical cancer. Future studies with larger sample sizes and multi-level validation are needed to confirm our findings and enhance their translational potential.

SPP1, located at position 4q13, with seven exons and six introns, is a member of the small integrin-binding ligand N-linked glycoprotein (SIBLING) family, activating matrix metalloproteinases (MMPs) involved in cancer development through extracellular matrix (ECM) degradation, angiogenesis, apoptosis, and soft tissue production (Trojanek, 2012; Kurnia et al., 2022). SPP1 is overexpressed in various types of cancer; however, research on its relationship with CC is limited (Xu C. et al., 2017; Choe et al., 2018; Zhang et al., 2020). Zhao et al. discovered that SPP1 expression was higher in CC tissues than in normal cervical epithelial tissues and significantly correlated with poor prognosis and immune cell infiltration, suggesting that SPP1 is a promising prognostic biomarker for CC patients (Zhao et al., 2021). The expression levels of RNF2 and SPP1 did not show significant differences between the tumor and normal groups in the RT-qPCR validation. This discrepancy could be attributed to several factors. First, RNF2 and SPP1 may undergo post-transcriptional regulation, which could affect their mRNA stability or translation efficiency, leading to discrepancies between mRNA and protein levels. Second, the functional effects of these genes might be more pronounced at the protein level. Post-translational modifications or protein degradation rates could influence their biological roles without corresponding changes in mRNA expression. Third, the limited number of samples in the RT-qPCR validation might have reduced the statistical power to detect significant differences. Future studies should expand the sample size to further validate these findings. Additionally, protein-level analyses, such as Western blot or immunohistochemistry (IHC), are recommended to confirm the expression levels of RNF2 and SPP1 in cervical cancer tissues. These protein-level validations will provide a more comprehensive understanding of the biological roles of these genes in cervical cancer. MMP1, produced by tumor cells, facilitates the hematogenous spread of squamous cell carcinoma (SCC) by inducing vascular permeability through endothelial protease-activated receptor (PAR)-1, aiding invasion and metastasis. LN metastasis in CC indicates a poor prognosis and is crucial for adjuvant therapy decision-making (Kurnia et al., 2022).

As a member of the MMP family, MMP1 directly facilitates tumor cell invasion and distant metastasis by degrading ECM and basement membrane components (Mao et al., 2022). Multiple studies have demonstrated that MMP1 is overexpressed in various malignancies and is associated with poor prognosis (Kurnia et al., 2022; Mao et al., 2022; Zhang W. et al., 2022). Notably, in cervical cancer, its elevated expression exhibits a significant correlation with tumor invasion and metastasis (Solovyeva et al., 2021). It is important to emphasize that the expression and activity of MMP1 are precisely regulated by the ubiquitination signaling network. Ubiquitin modification directly participates in regulating MMP1 expression by influencing the activation status of the NF-κB signaling pathway (Wu et al., 2014), which aligns closely with the ubiquitination-related regulatory network central to this study. Furthermore, IL-8 (CXCL8) can further upregulate MMP1 expression by activating the STAT3 signaling pathway, thereby enhancing tumor cell invasiveness (Chen et al., 2024). This suggests that MMP1 may act synergistically with CXCL8 to collectively drive the malignant progression of cervical cancer. In summary, MMP1 plays a pivotal role in the initiation and progression of cervical cancer, with its expression and activity modulated by multiple mechanisms, including the ubiquitination pathway. Therefore, in-depth investigation into the regulatory mechanisms of MMP1 provides a crucial theoretical foundation for developing therapeutic strategies targeting MMP1 and its upstream regulatory pathways.CXCL8 upregulation is associated with increased cancer risk and unfavorable prognosis in both lung cancer and CC (Pine et al., 2011). Its expression and function are cooperatively regulated by the ubiquitination pathway and multiple signaling pathways. On the one hand, E3 ubiquitin ligases can target the mRNA-binding proteins of CXCL8, promoting their ubiquitination-mediated degradation and thereby directly suppressing CXCL8 expression levels (Hu et al., 2025). On the other hand, ubiquitin modification indirectly influences the expression of CXCL8 and its pro-tumorigenic functions through the activation status of the MAPK/ERK signaling pathway (Jia et al., 2018). Additionally, E3 ubiquitin ligases can also impact the activation of the PI3K/AKT pathway. This effect synergizes with the pathway’s activation by CXCL8 itself, collectively promoting the survival and proliferation of cervical cancer cells by inhibiting apoptosis (Li et al., 2012). Simultaneously, CXCL8 may participate in inflammatory responses and immune regulation through activation of the NF-κB pathway, further driving tumor progression by promoting the production of pro-inflammatory cytokines (Zhang JY. et al., 2022).CXCL8 overexpression in tumor tissues correlates with bone metastasis in breast cancer patients. However, the role of CXCL8 in CC has not been ascertained (Bălăşoiu et al., 2014). Using a microarray dataset, Yan et al. discovered a significant upregulation of CXCL8 in CC tissues relative to normal tissues (Yan et al., 2017). They demonstrated a robust correlation between CXCL8 protein expression and the clinical stage, histological type, distant metastasis, and grade. Our study also observed higher CXCL8 expression in high-risk patients, with considerable variation in survival rates between the high- and low-risk groups.

Transferrin receptor 1 (TFRC), a pivotal regulator of iron metabolism, plays a significant role in the initiation and development of cervical cancer. Its elevated expression is not only significantly associated with advanced cancer stage, tumor stage, and lymph node metastasis but also serves as an indicator of poor prognosis for overall survival (OS) (Huang et al., 2022). Research indicates that TFRC directly promotes cervical carcinogenesis by participating in the regulation of the HIF-1 signaling pathway (Xu et al., 2019). Simultaneously, it promotes the activation of the PI3K/AKT/mTOR signaling pathway, thereby driving tumor cell proliferation and growth (Yang et al., 2025). Notably, the ubiquitination pathway, acting as a crucial regulatory hub, may modulate TFRC-involved iron homeostasis balance and associated cancer pathways through mechanisms such as targeted protein degradation, consequently influencing the cellular biological behaviors of cervical cancer (Yuan et al., 2023; Wang et al., 2022). In conclusion, the initiation and progression of cervical cancer constitute a complex process. TFRC, as a key molecule in iron metabolism, participates through the ubiquitination pathway in regulating multiple signaling pathways and cellular processes (Shang et al., 2024; Wang et al., 2025). In-depth investigation into the ubiquitination-mediated regulatory mechanisms of TFRC will contribute to the development of more effective therapeutic strategies for cervical cancer.

RNF2, a key E3 ubiquitin ligase within the RING finger protein family (An et al., 2018), exhibits upregulated expression in various cancers including breast cancer, colorectal cancer, and gastric cancer, and is closely associated with tumor initiation and progression (Zhang et al., 2017; We et al., 2020). In cervical cancer, RNF2 may affect cell cycle progression and promote cancer cell proliferation by facilitating the ubiquitin-mediated degradation of key regulatory proteins involved in the cell cycle (Yan et al., 2021). Simultaneously, as a core component of PRC1, RNF2 can suppress the expression of tumor suppressor genes through histone modifications, thereby further driving tumor development (Wang et al., 2015). Furthermore, RNF2 can stabilize ERα protein, regulating the progression of breast cancer; this mechanism may also function similarly in cervical cancer (Yuan et al., 2023). Notably, knocking down RNF2 enhances radiosensitivity and induces apoptosis in lung squamous cell carcinoma cells (Yang et al., 2019), suggesting that RNF2 may reduce the sensitivity of cervical cancer cells to radiotherapy and chemotherapy by participating in the DNA damage repair pathway. On the other hand, RNF2 may promote the recruitment of immunosuppressive cells, such as MDSCs, and inhibit T cell activation (Liang et al., 2025), thereby regulating the immune escape capability of cervical cancer cells. In summary, RNF2 likely plays a significant role in the proliferation, resistance to radiotherapy and chemotherapy, and immune evasion of cervical cancer, positioning it as a potential therapeutic target. In-depth investigation into the mechanisms of RNF2 in cervical cancer will provide novel insights and approaches for its treatment.

The findings of the present study on TFRC and CC align with those of previous studies that introduced the relationship between RNF2 expression and CC. Given the significance of radiotherapy in CC treatment, future studies should explore the association between these genes and radiotherapy sensitivity in CC (Franc et al., 2022). Survival and ROC curve analyses of the testing and validation set GSE52903 demonstrated that the identified biomarkers have strong diagnostic capabilities for identifying CC patients with a poor prognosis (Xu et al., 2023).While our model demonstrates promising predictive potential, it is important to acknowledge that the AUC values of the ROC curves are moderate, ranging from 0.6 to 0.7. This indicates that the model’s predictive power is not yet sufficient for clinical application without further refinement and validation. Future research should focus on improving the model’s accuracy by incorporating additional biomarkers and clinical variables.

KEGG analysis highlighted significant enrichment of the IL-17 and TNF signaling pathways in both the high- and low-risk subgroups, corroborating the recognized importance of the IL-17 pathway in CC (Li et al., 2019). Upregulation of the IL-17 signaling pathway facilitates the recruitment of suppressor cells derived from myeloid tissue, promoting angiogenesis, and inhibiting anti-tumor immunity (Holdbrooks et al., 2018). Tumor necrosis factor (TNF), known for its anti-tumor activity in cancer cells, plays a role in inflammation, differentiation, proliferation, and apoptosis as a member of the TNF cytokine superfamily. Specifically, TNF has been linked to CC, with activated TNF signaling pathways observed in tissues and cells (Xu L. et al., 2017). Li et al. confirmed the involvement of the TNF pathway in CC cells via a targeted relationship between miR195-5p and matrix metalloproteinase 14 (MMP14) (Li et al., 2018), suggesting that these pathways play a pivotal role in CC development and progression, warranting further investigation.

The TME is fertile ground for cancer cell development, with immune cell infiltration affecting tumor progression and therapeutic efficacy (Casey et al., 2015). These immune cells display dual characteristics; while some inhibit tumor development, others may encourage tumor growth. Our study identified three differentially expressed immune cell types (memory B cells, Macrophages M0, and neutrophils) in the high-risk group (Terlizzi et al., 2014)]. In contrast, Su et al. (Su et al., 2022)found that specific immune cells were correlated with prolonged OS in CC. In contrast, Yu et al. (Yu et al., 2021) observed a positive association between a high score and tumor-infiltrating immune cells. The absence of a similar relationship in our study may stem from differing analytical approaches and specimen types. Our results underscore the crucial roles of immune cell infiltration and immune-checkpoint gene expression in cervical cancer prognosis. These findings not only offer new insights into the immunological microenvironment of cervical cancer but may also aid in the development of novel therapeutic strategies, particularly for patients with elevated expression of immune-checkpoint genes.

Additionally, the blockade of immune checkpoints, such as PD-1/PD-L1 and CTLA-4, has emerged as a popular approach for treating malignant tumors. As the immune components of the TME often weaken anti-tumor responses, leading to limited success of immunotherapy as a monotherapy, identifying superior biomarkers and investigating combination therapies are essential for enhancing immunotherapy efficacy. Our study revealed elevated expression of TIGIT, LAG-3, GAL9, and PD-1 in low-risk subgroup—a finding seemingly contradictory to canonical views linking checkpoint markers to T-cell dysfunction and adverse outcomes (Ma et al., 2019; Josefsson et al., 2019). However, emerging evidence suggests immune checkpoints exhibit dual context-dependent roles: while chronic expression drives exhaustion, transient upregulation can signify active anti-tumor immunity (Kagabu et al., 2020). For instance, PD-1/LAG-3 are often co-expressed on tumor-infiltrating lymphocytes (TILs) in “inflamed” tumors, where pre-existing immunity is physiologically regulated rather than suppressed (Mollavelioglu et al., 2022). This aligns with our observations of concurrent CD4^+^ T-cell and memory B-cell infiltration in low-risk patients, implying a functional immune microenvironment poised for response. In summary, immune checkpoint molecules such as TIGIT, LAG-3, and PD-1 hold significant promise for application in tumor immunotherapy. By detecting the expression levels of these molecules, combined with other biomarkers and patient clinical information, personalized immunotherapy strategies can be guided, enhancing treatment efficacy and safety. Future research should further explore the predictive value of these markers across different tumor types and develop more effective combination treatment regimens.

In the present study, we performed a detailed examination of ubiquitin ligase genes in CC to enrich our understanding of their clinical relevance and uncover the molecular mechanisms that they influence. These ubiquitin ligase genes have the potential to serve as clinical biomarkers or therapeutic targets. We formulated a prognostic model to evaluate the clinical outcomes for CC patients. This model, which exhibits a strong predictive accuracy, identifies patients who are at a greater risk of recurrence, thereby assisting in the development of treatment strategies.

Firstly, the small sample size of this study may compromise statistical power and limit the robustness and generalizability of the results. Secondly, the lack of comprehensive clinical data for patients in the TCGA cohort poses challenges for in - depth analysis of clinical and pathological features in cervical cancer patients. In addition, the model development and validation of this study rely on retrospective analysis, so the clinical translation value of its conclusions needs further confirmation through prospective clinical trials. Finally, the precise molecular regulatory mechanisms and specific biological functions of ubiquitin - ligase genes in cervical cancer remain to be fully elucidated. In the future, we plan to expand the sample size through multi - center collaboration to enhance statistical power and verify the universality of the results. We will supplement experiments such as Western blotting and immunohistochemistry to validate the expression pattern of target genes at the protein level. Flow cytometry will be added to clarify the functional status of T cells (e.g., activation markers and cytokine secretion), providing direct functional evidence for the immune microenvironment conclusions. We will also conduct ubiquitination - related functional experiments (e.g., co - immunoprecipitation) to explore the role of these genes in the ubiquitination regulatory network and their specific mechanisms in cervical cancer progression, thereby offering a more solid theoretical basis and experimental support for prognosis prediction and targeted therapy in cervical cancer.

## 5 Conclusion

The five ubiquitination-related biomarkers (MMP1, RNF2, among others) identified herein have potential clinical applications in the diagnosis, prognosis, and therapeutic targeting of cervical cancer. These biomarkers not only contribute to a better understanding of the molecular mechanisms underlying cervical cancer but also inform the development of personalized treatment strategies, particularly in the analysis of sensitivity to immunotherapy and chemotherapeutic drugs. Future research should explore the application of these biomarkers in clinical practice to improve treatment outcomes in CC patients.

## Data Availability

The datasets presented in this study can be found in online repositories. The names of the repository/repositories and accession number(s) can be found in the article/Supplementary Material.
