# Supplementary figures and images for: Identification and validation of ubiquitination-related genes for predicting cervical cancer outcome
Source: Front Genet. 2025 Jul 30;16:1578075. doi: 10.3389/fgene.2025.1578075 (PMC12343280; doi:10.3389/fgene.2025.1578075)

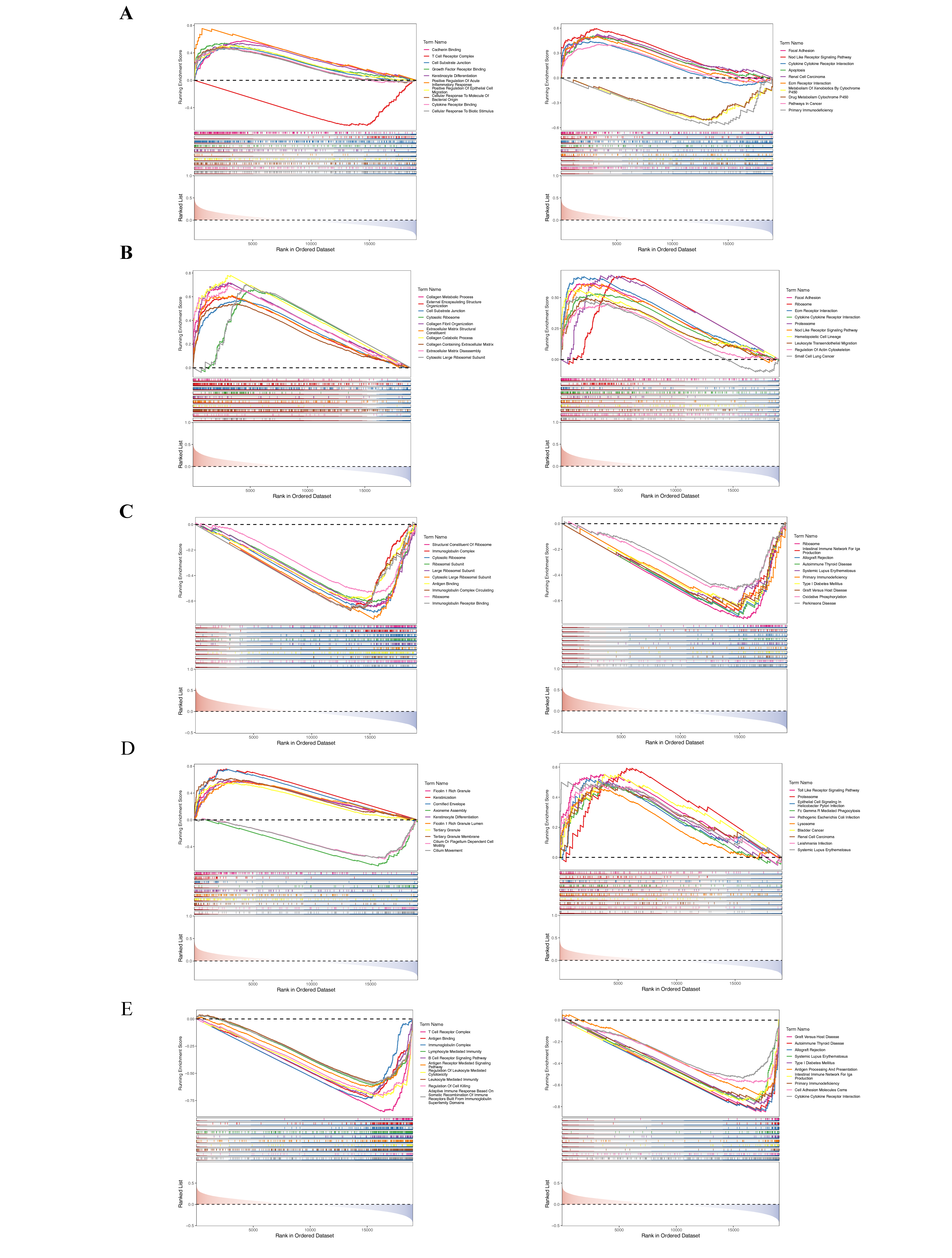

Supplement: Supplementary file 4 [file Image1.tif]
